# Supplementary material for: Altered microRNA Transcriptome in Cultured Human Liver Cells upon Infection with Ebola Virus
Source: Int J Mol Sci. 2021 Apr 6;22(7):3792. doi: 10.3390/ijms22073792 (PMC8038836; doi:10.3390/ijms22073792)
Supplement: Supplementary file 1 [file ijms-22-03792-s001.zip › Supplementary_File/D_ KEGG_Analysis_Results/16-30nt_pathway_Reston-24h-Huh7_vs_Control-24h-Huh7_up.mature_mirna_targets/hsa_pathwayResult.html]

| PathwayID | Definition | OriginalWebSite | Fisher-Pvalue | SelectionCounts | SelectionSize | Count | Size | FDR | Enrichment\_Score | GeneRatio | Genes |
| --- | --- | --- | --- | --- | --- | --- | --- | --- | --- | --- | --- |
| hsa05200 | Pathways in cancer - Homo sapiens (human) | http://www.genome.jp/kegg-bin/show\_pathway?hsa05200+26060+367+805+836+867+1021+1399+1438+28514+1906+54583+112399+2099+7976+8323+1647+2939+2947+3439+3458+3479+3558+3567+4040+4233+4257+5291+5468+9252+6500+6655+7048+7423+7476 | 9.942311e-05 | 34 | 258 | 529 | 7906 | 1.730419e-02 | 4.002513 | 0.131783 | APPL1//AR//CALM2//CASP3//CBL//CDK6//CRKL//CSF2RA//DLL1//EDN1//EGLN1//EGLN3//ESR1//FZD3//FZD6//GADD45A//GSTA2//GSTM3//IFNA1//IFNG//IGF1//IL2//IL5//LRP6//MET//MGST1//PIK3CB//PPARG//RPS6KA5//SKP1//SOS2//TGFBR2//VEGFB//WNT7A |
| hsa04115 | p53 signaling pathway - Homo sapiens (human) | http://www.genome.jp/kegg-bin/show\_pathway?hsa04115+545+836+1021+9538+1647+3479+4194+6241+10572+64393 | 1.036179e-04 | 10 | 258 | 72 | 7906 | 1.730419e-02 | 3.984565 | 0.038760 | ATR//CASP3//CDK6//EI24//GADD45A//IGF1//MDM4//RRM2//SIVA1//ZMAT3 |
| hsa04150 | mTOR signaling pathway - Homo sapiens (human) | http://www.genome.jp/kegg-bin/show\_pathway?hsa04150+6249+7976+8323+3479+8649+4040+5291+5562+58528+6655+7124+7248+9706+7476 | 4.537744e-04 | 14 | 258 | 153 | 7906 | 4.148630e-02 | 3.343160 | 0.054264 | CLIP1//FZD3//FZD6//IGF1//LAMTOR3//LRP6//PIK3CB//PRKAA1//RRAGD//SOS2//TNF//TSC1//ULK2//WNT7A |
| hsa04660 | T cell receptor signaling pathway - Homo sapiens (human) | http://www.genome.jp/kegg-bin/show\_pathway?hsa04660+925+3458+3558+3567+4690+56924+5291+5532+5535+6655+7124 | 5.048300e-04 | 11 | 258 | 103 | 7906 | 4.148630e-02 | 3.296855 | 0.042636 | CD8A//IFNG//IL2//IL5//NCK1//PAK6//PIK3CB//PPP3CB//PPP3R2//SOS2//TNF |
| hsa04010 | MAPK signaling pathway - Homo sapiens (human) | http://www.genome.jp/kegg-bin/show\_pathway?hsa04010+55799+836+1399+1946+2069+1647+3479+3556+8649+4214+4233+4615+4908+5532+5535+9693+9252+6655+7048+7124+7423 | 6.210524e-04 | 21 | 258 | 295 | 7906 | 4.148630e-02 | 3.206872 | 0.081395 | CACNA2D3//CASP3//CRKL//EFNA5//EREG//GADD45A//IGF1//IL1RAP//LAMTOR3//MAP3K1//MET//MYD88//NTF3//PPP3CB//PPP3R2//RAPGEF2//RPS6KA5//SOS2//TGFBR2//TNF//VEGFB |
| hsa05225 | Hepatocellular carcinoma - Homo sapiens (human) | http://www.genome.jp/kegg-bin/show\_pathway?hsa05225+1021+7976+8323+1647+2939+2947+4040+4233+4257+5291+6603+6655+7048+7476 | 1.150491e-03 | 14 | 258 | 168 | 7906 | 6.404401e-02 | 2.939117 | 0.054264 | CDK6//FZD3//FZD6//GADD45A//GSTA2//GSTM3//LRP6//MET//MGST1//PIK3CB//SMARCD2//SOS2//TGFBR2//WNT7A |
| hsa05418 | Fluid shear stress and atherosclerosis - Homo sapiens (human) | http://www.genome.jp/kegg-bin/show\_pathway?hsa05418+90+659+805+6347+1906+2939+2947+3458+4257+5291+5562+7124 | 1.898391e-03 | 12 | 258 | 139 | 7906 | 9.058038e-02 | 2.721614 | 0.046512 | ACVR1//BMPR2//CALM2//CCL2//EDN1//GSTA2//GSTM3//IFNG//MGST1//PIK3CB//PRKAA1//TNF |
| hsa00983 | Drug metabolism - other enzymes - Homo sapiens (human) | http://www.genome.jp/kegg-bin/show\_pathway?hsa00983+51727+2939+2947+3251+3614+4257+9+6241 | 4.063543e-03 | 8 | 258 | 79 | 7906 | 1.508026e-01 | 2.391095 | 0.031008 | CMPK1//GSTA2//GSTM3//HPRT1//IMPDH1//MGST1//NAT1//RRM2 |
| hsa03018 | RNA degradation - Homo sapiens (human) | http://www.genome.jp/kegg-bin/show\_pathway?hsa03018+694+57472+246175+29883+167227+1656+140886+255967 | 4.063543e-03 | 8 | 258 | 79 | 7906 | 1.508026e-01 | 2.391095 | 0.031008 | BTG1//CNOT6//CNOT6L//CNOT7//DCP2//DDX6//PABPC5//PAN3 |
| hsa04550 | Signaling pathways regulating pluripotency of stem cells - Homo sapiens (human) | http://www.genome.jp/kegg-bin/show\_pathway?hsa04550+90+659+7976+8323+3479+3625+3720+4762+84333+5291+7476 | 5.953697e-03 | 11 | 258 | 140 | 7906 | 1.629511e-01 | 2.225213 | 0.042636 | ACVR1//BMPR2//FZD3//FZD6//IGF1//INHBB//JARID2//NEUROG1//PCGF5//PIK3CB//WNT7A |
| hsa05014 | Amyotrophic lateral sclerosis (ALS) - Homo sapiens (human) | http://www.genome.jp/kegg-bin/show\_pathway?hsa05014+836+4842+5532+5535+6647+7124 | 5.990221e-03 | 6 | 258 | 51 | 7906 | 1.629511e-01 | 2.222557 | 0.023256 | CASP3//NOS1//PPP3CB//PPP3R2//SOD1//TNF |
| hsa04360 | Axon guidance - Homo sapiens (human) | http://www.genome.jp/kegg-bin/show\_pathway?hsa04360+659+1808+1946+55740+7976+4233+4690+56924+5291+5532+5535+9353+7222 | 6.195661e-03 | 13 | 258 | 181 | 7906 | 1.629511e-01 | 2.207912 | 0.050388 | BMPR2//DPYSL2//EFNA5//ENAH//FZD3//MET//NCK1//PAK6//PIK3CB//PPP3CB//PPP3R2//SLIT2//TRPC3 |
| hsa04012 | ErbB signaling pathway - Homo sapiens (human) | http://www.genome.jp/kegg-bin/show\_pathway?hsa04012+685+867+1399+2069+4690+56924+5291+6655 | 6.342409e-03 | 8 | 258 | 85 | 7906 | 1.629511e-01 | 2.197746 | 0.031008 | BTC//CBL//CRKL//EREG//NCK1//PAK6//PIK3CB//SOS2 |
| hsa05211 | Renal cell carcinoma - Homo sapiens (human) | http://www.genome.jp/kegg-bin/show\_pathway?hsa05211+1399+54583+112399+4233+56924+5291+6655 | 6.984662e-03 | 7 | 258 | 69 | 7906 | 1.666341e-01 | 2.155855 | 0.027132 | CRKL//EGLN1//EGLN3//MET//PAK6//PIK3CB//SOS2 |
| hsa04060 | Cytokine-cytokine receptor interaction - Homo sapiens (human) | http://www.genome.jp/kegg-bin/show\_pathway?hsa04060+90+651+659+6347+56477+6355+1230+1438+3439+3458+3556+3558+53832+56300+3567+3625+7048+7124 | 7.503828e-03 | 18 | 258 | 294 | 7906 | 1.670852e-01 | 2.124717 | 0.069767 | ACVR1//BMP3//BMPR2//CCL2//CCL28//CCL8//CCR1//CSF2RA//IFNA1//IFNG//IL1RAP//IL2//IL20RA//IL36G//IL5//INHBB//TGFBR2//TNF |
| hsa05224 | Breast cancer - Homo sapiens (human) | http://www.genome.jp/kegg-bin/show\_pathway?hsa05224+1021+28514+2099+7976+8323+1647+3479+4040+5291+6655+7476 | 8.509531e-03 | 11 | 258 | 147 | 7906 | 1.748964e-01 | 2.070094 | 0.042636 | CDK6//DLL1//ESR1//FZD3//FZD6//GADD45A//IGF1//LRP6//PIK3CB//SOS2//WNT7A |
| hsa04114 | Oocyte meiosis - Homo sapiens (human) | http://www.genome.jp/kegg-bin/show\_pathway?hsa04114+367+805+996+64506+132864+22849+3479+5532+5535+6500 | 8.901914e-03 | 10 | 258 | 128 | 7906 | 1.748964e-01 | 2.050517 | 0.038760 | AR//CALM2//CDC27//CPEB1//CPEB2//CPEB3//IGF1//PPP3CB//PPP3R2//SKP1 |
| hsa04658 | Th1 and Th2 cell differentiation - Homo sapiens (human) | http://www.genome.jp/kegg-bin/show\_pathway?hsa04658+28514+3458+3558+3567+4094+5532+5535+864 | 1.010029e-02 | 8 | 258 | 92 | 7906 | 1.874165e-01 | 1.995666 | 0.031008 | DLL1//IFNG//IL2//IL5//MAF//PPP3CB//PPP3R2//RUNX3 |
| hsa05220 | Chronic myeloid leukemia - Homo sapiens (human) | http://www.genome.jp/kegg-bin/show\_pathway?hsa05220+867+1021+1399+1647+5291+6655+7048 | 1.168324e-02 | 7 | 258 | 76 | 7906 | 2.053791e-01 | 1.932437 | 0.027132 | CBL//CDK6//CRKL//GADD45A//PIK3CB//SOS2//TGFBR2 |
| hsa04710 | Circadian rhythm - Homo sapiens (human) | http://www.genome.jp/kegg-bin/show\_pathway?hsa04710+8553+79365+5562+6500 | 1.743486e-02 | 4 | 258 | 31 | 7906 | 2.911622e-01 | 1.758582 | 0.015504 | BHLHE40//BHLHE41//PRKAA1//SKP1 |
| hsa05144 | Malaria - Homo sapiens (human) | http://www.genome.jp/kegg-bin/show\_pathway?hsa05144+6347+3458+4233+4615+7124 | 2.107663e-02 | 5 | 258 | 49 | 7906 | 3.319134e-01 | 1.676199 | 0.019380 | CCL2//IFNG//MET//MYD88//TNF |
| hsa05210 | Colorectal cancer - Homo sapiens (human) | http://www.genome.jp/kegg-bin/show\_pathway?hsa05210+26060+836+2069+1647+5291+6655+7048 | 2.186256e-02 | 7 | 258 | 86 | 7906 | 3.319134e-01 | 1.660299 | 0.027132 | APPL1//CASP3//EREG//GADD45A//PIK3CB//SOS2//TGFBR2 |
| hsa04650 | Natural killer cell mediated cytotoxicity - Homo sapiens (human) | http://www.genome.jp/kegg-bin/show\_pathway?hsa04650+836+3439+3458+5291+5532+5535+135250+6655+7124 | 2.724278e-02 | 9 | 258 | 131 | 7906 | 3.690542e-01 | 1.564749 | 0.034884 | CASP3//IFNA1//IFNG//PIK3CB//PPP3CB//PPP3R2//RAET1E//SOS2//TNF |
| hsa04668 | TNF signaling pathway - Homo sapiens (human) | http://www.genome.jp/kegg-bin/show\_pathway?hsa04668+836+6347+1051+1906+3659+5291+9252+7124 | 2.971505e-02 | 8 | 258 | 112 | 7906 | 3.690542e-01 | 1.527024 | 0.031008 | CASP3//CCL2//CEBPB//EDN1//IRF1//PIK3CB//RPS6KA5//TNF |
| hsa05143 | African trypanosomiasis - Homo sapiens (human) | http://www.genome.jp/kegg-bin/show\_pathway?hsa05143+3458+4615+7064+7124 | 3.146056e-02 | 4 | 258 | 37 | 7906 | 3.690542e-01 | 1.502234 | 0.015504 | IFNG//MYD88//THOP1//TNF |
| hsa04657 | IL-17 signaling pathway - Homo sapiens (human) | http://www.genome.jp/kegg-bin/show\_pathway?hsa04657+836+6347+1051+8061+3458+3567+7124 | 3.188039e-02 | 7 | 258 | 93 | 7906 | 3.690542e-01 | 1.496476 | 0.027132 | CASP3//CCL2//CEBPB//FOSL1//IFNG//IL5//TNF |
| hsa05163 | Human cytomegalovirus infection - Homo sapiens (human) | http://www.genome.jp/kegg-bin/show\_pathway?hsa05163+805+836+6347+1230+1021+1399+3439+5291+5532+5535+6655+7124+7248 | 3.252025e-02 | 13 | 258 | 225 | 7906 | 3.690542e-01 | 1.487846 | 0.050388 | CALM2//CASP3//CCL2//CCR1//CDK6//CRKL//IFNA1//PIK3CB//PPP3CB//PPP3R2//SOS2//TNF//TSC1 |
| hsa04350 | TGF-beta signaling pathway - Homo sapiens (human) | http://www.genome.jp/kegg-bin/show\_pathway?hsa04350+90+659+3458+3625+6500+7048+7124 | 3.352601e-02 | 7 | 258 | 94 | 7906 | 3.690542e-01 | 1.474618 | 0.027132 | ACVR1//BMPR2//IFNG//INHBB//SKP1//TGFBR2//TNF |
| hsa05330 | Allograft rejection - Homo sapiens (human) | http://www.genome.jp/kegg-bin/show\_pathway?hsa05330+3458+3558+3567+7124 | 3.429064e-02 | 4 | 258 | 38 | 7906 | 3.690542e-01 | 1.464824 | 0.015504 | IFNG//IL2//IL5//TNF |
| hsa00480 | Glutathione metabolism - Homo sapiens (human) | http://www.genome.jp/kegg-bin/show\_pathway?hsa00480+124975+2939+2947+4257+6241 | 3.518796e-02 | 5 | 258 | 56 | 7906 | 3.690542e-01 | 1.453606 | 0.019380 | GGT6//GSTA2//GSTM3//MGST1//RRM2 |
| hsa05214 | Glioma - Homo sapiens (human) | http://www.genome.jp/kegg-bin/show\_pathway?hsa05214+805+1021+1647+3479+5291+6655 | 3.520107e-02 | 6 | 258 | 75 | 7906 | 3.690542e-01 | 1.453444 | 0.023256 | CALM2//CDK6//GADD45A//IGF1//PIK3CB//SOS2 |
| hsa04218 | Cellular senescence - Homo sapiens (human) | http://www.genome.jp/kegg-bin/show\_pathway?hsa04218+545+805+1021+1647+5291+5532+5535+144715+7048+7248 | 3.632896e-02 | 10 | 258 | 160 | 7906 | 3.690542e-01 | 1.439747 | 0.038760 | ATR//CALM2//CDK6//GADD45A//PIK3CB//PPP3CB//PPP3R2//RAD9B//TGFBR2//TSC1 |
| hsa04614 | Renin-angiotensin system - Homo sapiens (human) | http://www.genome.jp/kegg-bin/show\_pathway?hsa04614+186+57486+7064 | 3.756839e-02 | 3 | 258 | 23 | 7906 | 3.690542e-01 | 1.425177 | 0.011628 | AGTR2//NLN//THOP1 |
| hsa04964 | Proximal tubule bicarbonate reclamation - Homo sapiens (human) | http://www.genome.jp/kegg-bin/show\_pathway?hsa04964+2747+4190+8671 | 3.756839e-02 | 3 | 258 | 23 | 7906 | 3.690542e-01 | 1.425177 | 0.011628 | GLUD2//MDH1//SLC4A4 |
| hsa00564 | Glycerophospholipid metabolism - Homo sapiens (human) | http://www.genome.jp/kegg-bin/show\_pathway?hsa00564+1040+8526+160851+2819+2820+253558+81579 | 3.879965e-02 | 7 | 258 | 97 | 7906 | 3.702595e-01 | 1.411172 | 0.027132 | CDS1//DGKE//DGKH//GPD1//GPD2//LCLAT1//PLA2G12A |
| hsa05170 | Human immunodeficiency virus 1 infection - Homo sapiens (human) | http://www.genome.jp/kegg-bin/show\_pathway?hsa05170+545+805+836+1399+3439+4615+56924+5291+5532+5535+6500+7124 | 4.454183e-02 | 12 | 258 | 212 | 7906 | 3.919255e-01 | 1.351232 | 0.046512 | ATR//CALM2//CASP3//CRKL//IFNA1//MYD88//PAK6//PIK3CB//PPP3CB//PPP3R2//SKP1//TNF |
| hsa04061 | Viral protein interaction with cytokine and cytokine receptor - Homo sapiens (human) | http://www.genome.jp/kegg-bin/show\_pathway?hsa04061+6347+56477+6355+1230+3558+53832+7124 | 4.459032e-02 | 7 | 258 | 100 | 7906 | 3.919255e-01 | 1.350759 | 0.027132 | CCL2//CCL28//CCL8//CCR1//IL2//IL20RA//TNF |
| hsa04933 | AGE-RAGE signaling pathway in diabetic complications - Homo sapiens (human) | http://www.genome.jp/kegg-bin/show\_pathway?hsa04933+836+6347+1906+5291+7048+7124+7423 | 4.459032e-02 | 7 | 258 | 100 | 7906 | 3.919255e-01 | 1.350759 | 0.027132 | CASP3//CCL2//EDN1//PIK3CB//TGFBR2//TNF//VEGFB |
| hsa00563 | Glycosylphosphatidylinositol (GPI)-anchor biosynthesis - Homo sapiens (human) | http://www.genome.jp/kegg-bin/show\_pathway?hsa00563+5277+51227+94005 | 4.653521e-02 | 3 | 258 | 25 | 7906 | 3.985323e-01 | 1.332218 | 0.011628 | PIGA//PIGP//PIGS |
| hsa05142 | Chagas disease (American trypanosomiasis) - Homo sapiens (human) | http://www.genome.jp/kegg-bin/show\_pathway?hsa05142+6347+3458+3558+4615+5291+7048+7124 | 4.874451e-02 | 7 | 258 | 102 | 7906 | 4.070166e-01 | 1.312074 | 0.027132 | CCL2//IFNG//IL2//MYD88//PIK3CB//TGFBR2//TNF |
